# Research Domain Criteria and Deaths by Suicide in the National Violent Death Reporting System

**DOI:** 10.1001/jamanetworkopen.2026.4024

**Published:** 2026-03-30

**Authors:** Susan D. Cochran, Christina Chance, Alina Arseniev-Koehler, Bruce Cuthbert, Vickie M. Mays

**Affiliations:** 1 Department of Epidemiology, Fielding School of Public Health, and Department of Statistics and Data Science, University of California Los Angeles; 2 Department of Computer Science, Samueli School of Engineering, University of California Los Angeles; 3 Department of Sociology, Regenstrief Center for Healthcare Engineering, Purdue University, West Lafayette, Indiana; 4 Retired, formerly Research Domain Criteria Unit, National Institute of Mental Health, Bethesda, Maryland; 5 Department of Psychology, University of California Los Angeles; 6 Department of Health Policy and Management, Fielding School of Public Health, University of California Los Angeles

## Abstract

**Importance:**

Mental health morbidity is a proximal factor in suicide. Using the research domain criteria (RDoC) framework to investigate sex and age differences in psychopathology at the time of death may provide better characterization than focusing on clinical diagnosis alone.

**Objectives:**

To (1) evaluate whether token-based and large language model scoring of RDoC can be successfully applied to law enforcement and coroner or medical examiner death narratives in the US National Violent Death Reporting System (NVDRS), and (2) investigate sex and age differences in clinically relevant scores to illuminate underidentified dimensions of mental health dysfunction proximal to suicide.

**Design, Setting, and Participants:**

This cross-sectional study drew on death records in the restricted-access 2020 to 2021 NVDRS. Participants were limited to suicide decedents aged 12 years and older from all 50 states whose death record included a law enforcement and coroner or medical examiner death narrative of 20 words or more. Analyses were conducted between May 2024 and September 2025.

**Exposures:**

Sex and age of decedents.

**Main outcomes and measures:**

RDoC symptom scores; mental health status measures in death records (mental health diagnosis, current depressed mood, and alcohol or drug misuse).

**Results:**

Using both a token-based system and a large language model approach, law enforcement and coroner or medical examiner narratives of 72 585 suicide decedents were scored (mean [SD] age, 46.3 [19.3] years; 57 770 [80.6%] male). Both methods were previously validated with psychiatric electronic health records. To validate this approach, token density and large language model scores were compared with current NVDRS mental health status measures. Both scoring methods correlated with precoded measures and demonstrated levels of neurobehavioral dysfunction at the time of death similar to psychiatric inpatients on admission. Sex- and age-related differences in clinically relevant dysfunction showed the highest levels among female vs male and younger vs older decedents after adjusting for demographic confounding.

**Conclusions and relevance:**

This cross-sectional study of suicide decedents found that information relevant to RDoC domains is encoded in NVDRS death narratives and can be extracted using large language models. The approach used here observed more pervasive neurobehavioral dysfunction among suicide decedents than that captured by currently employed NVDRS measures of mental health.

## Key Points

**Question:**

Can large language models be used to assign research domain criteria (RDoC) scores to suicide death narratives so as to better understand sex- and age-associated psychiatric morbidity among decedents?

**Findings:**

Using cross-sectional information from 72 585 deaths by suicide in the 2020 to 2021 National Violent Death Reporting System (NVDRS), methods of large language models were applied to score text narratives for RDoC domains. Decedents exhibited clinically elevated scores across most domains, with female and younger decedents scoring higher than male and older decedents, respectively.

**Meaning:**

These findings suggest that, despite the relatively low prevalence of clinically depressed mood in NVDRS deaths by suicide, RDoC scores indicate substantial levels of clinically relevant symptoms at the time of death.

## Teaser

This cross-sectional study evaluates whether large language model scoring of research domain criteria can be successfully applied to law enforcement and coroner or medical examiner death narratives in the US National Violent Death Reporting System.

## Introduction

Suicide is a major contributor to early mortality, particularly among those 15 to 45 years of age^1^ where it is the second leading cause of death for men and fourth for women in the US.^2,3^ Both mental health and substance use disorders are well-known risk factors for suicide.^4^ Indeed, 80% of individuals who report a lifetime suicide attempt also meet criteria for a mental health disorder.^5^ In psychological autopsy studies^6,7^ more than 87% of deaths by suicide show evidence of a concurrent mental health disorder (primarily affective disorders) and/or a substance use disorder. Yet, estimates drawn from the nation’s major tracking source for violent death, the National Violent Death Reporting System (NVDRS), suggest that perhaps less than half of those who die by suicide have a mental health disorder at the time of their death, and less than a third are described as depressed.^4,8,9^ This lower-than-expected prevalence has been attributed to a possible undercount of mental health morbidity.^10^ As information within this system is critical to understanding precursors to suicide,^11^ undercounting the contributory role of psychopathology may reduce focus on a key precipitant.

There are at least 2 possible reasons for the undercount. One is that current methods fail to capture the nuances of symptom expression accompanying suicide,^12^ which might nevertheless be detectable using large language models.^13^ Each death incident within the NVDRS is a mixture of precoded variables, such as the presence of any diagnosed mental disorder, and 2 brief text narratives (law enforcement [LE] and coroner or medical examiner [CME]) summarizing proximal circumstances of the death. Although often underutilized, these narratives have been analyzed using topic modeling, to reveal, for example, gender differences in the expression of psychological distress that are not always aligned with diagnostic categories.^13^ A second reason is that the presence of psychopathology may be more readily detectable using the dimensional approach of research domain criteria (RDoC),^14^ especially given the type of information available to those who create the NVDRS death records. Records are compiled by trained state-level public health workers (PHWs) who use a detailed coding manual to summarize death certificates, LE reports, CME reports, and public records (eg, newspaper articles or social media). In contrast to psychiatric diagnostic systems that require specific symptom profiles, the RDoC framework offers a more holistic, descriptive approach to measuring neurobehavioral disturbances across 6 functional domains (negative valence, positive valence, social processes, arousal processes, cognitive systems, and sensorimotor systems)^14,15^ that may be more compatible with NVDRS construction. For example, hopelessness, a major predictor of suicide,^16^ reflects disruption in negative valence. In contrast, anxiety and agitation^17^ indicate disrupted arousal processes. Neither of these constructs is currently measured in the NVDRS but may appear in the narratives.

In the present study, we used the RDoC framework and text data drawn from the 2 NVDRS narratives to illuminate patterns of neurobehavioral dysfunction among suicide decedents. Our objectives were to characterize the extent of dysfunction while also focusing on possible sex^13^ and age^18,19^ differences. To do so, we applied 2 emerging methods^15,20,21^ in machine learning that have been used successfully with psychiatric inpatient electronic health records (EHR). One in particular, the prompting of large language models (LLMs), has been shown to capture the severity of psychopathology among psychiatric patients, even without special model training for psychiatric EHR data.^15,20,22^ Whether these approaches will work effectively with the 2 NVDRS text narratives has yet to be determined. If they do, they will offer a relatively straightforward method for investigating understudied aspects of mental health disturbance among suicide decedents.

## Methods

### Data Source

We used 2 years of the NVDRS (2020-2021) database, which is available from the Centers for Disease Control and Prevention.^9^ The NVDRS includes incident descriptions of violent deaths (eg, homicide and suicide) occurring within the US and its territories. In 2020 and 2021, the NVDRS recorded 79 933 suicide decedents aged 12 years and older, including 428 deaths from US territories, which we excluded. We preprocessed the 2 narratives accompanying the death incident (eg, lower-casing, spell checking, and acronym expansion). We then restricted our sample to incidents with at least 1 narrative containing 20 words or more to ensure the accuracy of text-based methods. The selection process is outlined the eFigure in Supplement 1). The study followed the Strengthening the Reporting of Observational Studies in Epidemiology (STROBE) reporting guideline and was deemed exempt from the need for approval and informed consent by the UCLA Office of Human Research Protection because research on deceased individuals is not considered human participants research.

### Measures

#### Demographic Characteristics

The NVDRS includes decedent’s sex (male or female) and age in years, as well as potential demographic confounders^8,13^: relationship status,^23^ which we recoded into 2 categories (married, cohabiting, or partnered and single or unknown), military veteran status (yes or no or unknown),^24^ US Census region^13^ (Northeast, Midwest, South, and West) and race and ethnicity recoded into 6 categories: Hispanic, non-Hispanic African American, non-Hispanic American Indian or Native American, non-Hispanic Asian American, non-Hispanic other or unknown, and non-Hispanic White.^13^

#### Mental Health Status

We used 4 indicators in the NVDRS to capture 3 aspects of mental health status. First, PHWs classified decedents as having a mental health disorder^25^ other than alcohol or substance use abuse or dependency (yes, no, or unknown). Coding instructions^26^ directed that only evidence of a current or past diagnosis and/or treatment not described as successful could be used. Second, although the NVDRS does not code substance dependency and/or abuse, we developed a proxy combining 2 items. One classified decedents for problematic alcohol use, addiction, and/or indications of alcohol-related treatment or rehabilitation; the other for problematic drug use and/or addiction, any illicit drug use, and/or drug use treatment. We coded decedents yes for problematic alcohol or drug use if either measure was PHW-coded yes, and no or unknown otherwise. Third, PHWs coded decedents for current depression at time of death, whether reported by the decedent or others (yes, no, or unknown). For this, the codebook restricted positive coding to the occurrence of specific words in records describing the decedent as “depressed, sad, despondent, down, blue, low, unhappy, etc.”^26^ Other words potentially signaling emotional distress were explicitly excluded, including “agitated, angry, mad, anxious, overwrought, etc.”^26^

#### RDoC Domain Scores

We used 2 methods to process narratives. First, we employed a token-based system developed by McCoy et al.^21^ This system scores 5 of the 6 RDoC domains (negative valence systems, positive valence systems, social processes, cognitive systems, and arousal processes) using counts of domain-specific tokens (individual or pairs of words) identified from a natural language processing (NLP) model trained on psychiatric patient records. Both their validation studies with psychiatric inpatients^15,21^ and later juvenile emergency department visits^20^ demonstrate that these token-based scores successfully predict length of hospital stay and inpatient admission. Positive valence scores obtained from discharge records also predicted readmission risk, consistent with substance use relapse. For the current study, we employed their token-based system to generate 5 domain-specific scores for each narrative. RDoC token density scores were calculated as the sum of domain-specific tokens present per 100 total narrative tokens.

Second, using a local instance of an LLM (Meta-Llama-3-8B-Instruct.Q4_K_M.gguf)^27^ obtained via HuggingFace,^28^ we estimated RDoC scores for all 6 RDoC domains following the approach of McCoy et al.^15,20^ We first determined that the LLM had been trained on the RDoC System by prompting it to describe the RDoC and its domains (see eTable 1 in Supplement 1). Then we estimated token scores for each of the 6 domains using a zero-shot system prompt modeled on McCoy et al:^15,20^ “You are a skilled NIMH Research Domain Criteria (RDoC) coder. Your task is to score a brief narrative from a postmortem review of a suicide on a single RDoC domain. Use ONLY the information within this narrative and your skill. Remember that substance use can be reflected as a positive valence system symptom. Score this narrative on a 0–10 scale capturing the magnitude of documented domain symptoms: 0 if no symptoms are present or functioning is normal, 1–3 for mild symptoms, 4–6 for moderate symptoms requiring treatment, 7–9 for severe symptoms requiring hospitalization, and 10 if symptoms are extremely severe” (see eTable 2 in Supplement 1). To generate LLM scores unaffected by model decisions for other domains or narratives, we conducted independent trials for each domain within each narrative, providing the LLM with the prompt, a single domain to be scored, and a corresponding narrative. After evaluating the information, the model produced a token score. Temperature for the model was set at 0 to ensure it would produce the most probable token given narrative input. To assess the adequacy of preexisting RDoC model training, we also calculated a perplexity score for the token by taking the exponent of its negative log probability.^29^ Perplexity scores range from 1 to infinity, with lower scores indicating greater certainty in the token generated. Finally, we calculated a total word count for each narrative.

For some analyses, we recoded RDoC domain scores to binomial distributions (any tokens present or an LLM score greater than 0 vs no tokens present or an LLM score of 0) to be consistent with the binomial PHW-coded measures. We also used the scoring approach of the LLM prompt to recode LLM RDoC scores into those having clinical relevance or not (a score of 4-10 indicating symptoms requiring treatment vs 0-3 indicating at most mild symptoms). Finally, we created a summary measure for both LE and CME narratives that categorized each death as either having any clinically relevant RDoC LLM score vs not.

### Statistical Analysis

Statistical analyses were conducted using Stata.^30^ We proceeded in 5 steps. First, missing data, though rare (4 deaths had missing age), were imputed. Second, we examined sex differences in demographic features and PHW-coded mental health indicators using logistic regression methods. We report adjusted odds ratios estimating female gender from demographic features and mental health indicators considered simultaneously. Third, we evaluated indicators of convergent validity. Using the Kendall tau-B statistic, we compared correlations between domain scores derived from the RDoC token method, which focuses on the presence of words or word phrases predictive of domain dysfunction, and the LLM prompt-based scoring method. Then, after dichotomizing RDoC measures to indicate the presence or absence of domain dysfunctions, we investigated associations between RDoC token density, LLM scores, and PHW-coded mental status indicators via Phi correlations. Fourth, we employed logistic regression methods to investigate age-related differences in PHW-coded indicators and RDoC scores, aiming to clarify age-related patterns after adjusting for demographic confounders. Fifth, we employed nested logistic regression methods, estimating 2 models, 1 being a restricted version of the other, to isolate the gain in model fit when using clinically relevant RDoC scores to predict sex differences. The first model characterized sex differences among decedents, after adjusting for demographic confounders and PHW-coded mental health markers. The second added clinically relevant RDoC domain scores. Incremental improvement between the 2 models was evaluated via likelihood ratio χ^2^ tests. For sex and age difference analyses, when evaluating the contributory benefits of LLM scores, we also adjusted for narrative word count. All findings were evaluated using α < .05. Analyses were conducted between May 2024 and September 2025 using Stata version 19.0 (StataCorp).

## Results

Among the 72 585 suicide decedents (5447 [7.5%] non-Hispanic African American, 5973 [8.2%] Hispanic, and 56 933 [78.4%] White), most were male (57 770 decedents [80.6%]), middle-aged (mean [SD] age, 46.3 [19.3] years), and not partnered (41 956 decedents [57.8%]). A total of 10 729 male decedents (18.6%) were military veterans, and about a third of all decedents lived in the South (23 127 decedents [31.9%]) (see Table 1). Evidence of any mental disorder (32 190 decedents [44.4%]) and/or problematic alcohol and/or drug use (19 935 decedents [27.5%]) was prevalent; however, only 20 256 decedents (27.9%) were classified as currently depressed. Male and female decedents differed significantly across most demographic and mental health status features, with female decedents more likely than male decedents to have a mental health problem and male decedents more likely to have an alcohol and/or drug use problem. Female decedents were no more likely than males to be depressed.

**Table 1.** Characteristics of Female and Male Suicide Decedents, 2020-2021 National Violent Death Reporting System (NVDRS)

Characteristic	Decedents, No. (%)			aOR (95% CI)^a^	*P* value
	Female (n = 14 815)	Male (n = 57 770)	Total (N = 72 585)		
Demographic characteristics					
Age, y					
12-24	2253 (15.2)	8439 (14.6)	10 692 (14.7)	0.95 (0.88-1.02)	.13
25-44	4984 (33.6)	20 242 (35.0)	25 226 (34.8)	0.92 (0.86-0.98)	.01
45-64	5482 (37.0)	17 899 (31.0)	23 381 (32.2)	1.15 (1.09-1.22)	<.001
≥65	2096 (14.2)	11 190 (19.4)	16 286 (18.3)	1 [Reference]	NA
Ethnicity and race					
Hispanic	1151 (7.8)	4822 (8.4)	5973 (8.2)	0.91 (0.84-0.97)	.01
Non-Hispanic African-American	1064 (7.2)	4383 (7.6)	5447 (7.5)	1.00 (0.93-1.08)	.89
Non-Hispanic American Indian and Alaska Native	250 (1.7)	694 (1.2)	944 (1.3)	1.56 (1.34-1.81)	<.001
Non-Hispanic Asian	624 (4.2)	1489 (2.6)	2113 (2.9)	1.50 (1.36-1.66)	<.001
Non-Hispanic other or unknown^b^	286 (1.9)	889 (1.5)	1175 (1.6)	1.27 (1.10-1.46)	.001
Non-Hispanic White	11 440 (77.2)	45 493 (78.8)	56 933 (78.4)	1 [Reference]	NA
Relationship status					
Married, cohabiting, or partnered	6478 (43.7)	24 151 (41.8)	30 629 (42.2)	1.13 (1.08-1.17)	<.001
Single or unknown	8337 (56.3)	33 619 (58.2)	41 956 (57.8)	1 [Reference]	NA
Military experience					
Veteran	462 (3.1)	10 729 (18.6)	11 191 (15.4)	0.14 (0.13-0.15)	<.001
Nonveteran or unknown	14 353 (96.9)	47 041 (81.4)	61 394 (84.6)	1 [Reference]	
Census region					
Midwest	3671 (24.8)	15 106 (26.2)	18 777 (25.9)	0.92 (0.86-0.97)	.01
South	4532 (30.6)	18 595 (32.2)	23 127 (31.9)	0.99 (0.94-1.05)	.76
West	4238 (28.6)	15 543 (26.9)	19 781 (27.2)	1.04 (0.98-1.10)	.17
Northeast	2374 (16.0)	8526 (14.8)	10 900 (15.0)	1 [Reference]	NA
Incident year					
2021	7665 (51.7)	30 015 (52.0)	37 680 (51.9)	0.98 (0.94-1.02)	.28
2020	7150 (48.3)	27 755 (48.0)	34 905 (48.1)	1 [Reference]	NA
Mental health measures					
Any mental health problem					
Yes	8787 (59.3)	23 403 (40.5)	32 190 (44.4)	2.19 (2.11-2.28)	<.001
No or unknown	6028 (40.7)	34 367 (59.5)	40 395 (55.6)	1 [Reference]	NA
Alcohol or drug problem					
Yes	3885 (26.2)	16 050 (27.8)	19 935 (27.5)	0.77 (0.73-0.80)	<.001
No or unknown	10 930 (73.8)	41 720 (72.2)	52 650 (72.5)	1 [Reference]	NA
Currently depressed					
Yes	4272 (28.8)	15 984 (27.7)	20 256 (27.9)	0.97 (0.93-1.01)	.11
No or unknown	10 543 (71.2)	41 786 (72.3)	52 329 (72.1)	1 [Reference]	NA

Abbreviations: aOR, adjusted odds ratio, NA, not applicable.

^a^ Adjusted odds ratio derived from logistic regression model characterizing female sex from all demographic characteristics and mental health measures considered simultaneously.

^b^ Decedent ethnicity and race classified as other or unknown includes 2 or more races specified non-Hispanic, other or unspecified non-Hispanic, and unknown race non-Hispanic.

RDoC scoring of the LE and CME narratives revealed similar patterns of elevation in domain dysfunctions using either method (see eTable 3 in Supplement 1). RDoC token density demonstrated the highest density for negative valence, positive valence, and arousal processes. Similarly, LLM-generated RDoC scores showed the highest scores for negative and positive valence. On average, LLM scores were consistent with severe dysfunctions in negative valence (mean [SD] LE, 7.32 [2.67]; CME, 7.15 [2.72]) and moderate dysfunction levels for arousal processes (mean [SD] LE, 4.01 [3.50]; CME, 4.01 [3.43]). Mild elevation was observed for both positive valence (mean [SD] LE, 2.11 [2.95]; CME, 2.18 [2.93]) and social processes (mean [SD] LE, 2.06 [2.84]; CME, 1.92 [2.78]).

As anticipated, scores from the 2 methods were positively correlated. In addition, RDoC token densities, and LLM scores even more so, positively covaried with the 3 indicators of mental health status, except for sensorimotor systems (see Table 2). The PHW-coded measures were themselves positively intercorrelated (mental health problem with alcohol or drug misuse: phi = 0.14; *P* < .001; depression: phi = 0.11; *P* < .001; alcohol or drug misuse with depression: phi = 0.05; *P* < .001).

**Table 2.** Associations Between Research Domain Criteria (RDoC) Tokens and Large Language Model (LLM) Scores, and National Violent Death Reporting System (NVDRS) Mental Health Measures

RDoC Domain	Phi correlation coefficient^a^					
	Any mental health problem		Alcohol or drug problem		Current depressed mood	
	RDoC tokens^b^	LLM score^c^	RDoC tokens^b^	LLM score^c^	RDoC tokens^b^	LLM score^c^
Law enforcement narrative (n = 51 240)						
Negative valence	0.14	0.11	0.07	0.08	0.12	0.12
Positive valence	0.15	0.19	0.31	0.28	0.08	0.14
Social processes	0.10	0.20	0.08	0.15	0.03	0.20
Arousal processes	0.11	0.22	0.06	0.21	0.04	0.17
Cognitive systems	0.11	0.2	0.05	0.05	0.02	0.13
Sensorimotor systems^d^	NA	−0.04	NA	−0.02	NA	−0.05
Coroner or medical examiner narrative (n = 68 655)						
Negative valence	0.10	0.20	0.04	0.14	0.13	0.13
Positive valence	0.13	0.21	0.32	0.36	0.08	0.10
Social processes	0.08	0.24	0.08	0.18	0.03	0.20
Arousal processes	0.07	0.29	0.05	0.28	0.04	0.15
Cognitive systems	0.09	0.24	0.04	0.08	0.03	0.12
Sensorimotor systems^d^	NA	−0.05	NA	−0.03	NA	−0.04

Abbreviation: NA, not applicable.

^a^ All *P* values were less than .001.

^b^ Token density recoded as any tokens present vs. none.

^c^ Score recoded as 1 to 10 vs 0.

^d^ Sensorimotor domain tokens not included in RDoC token system.

We then investigated patterns of RDoC dysfunction across the life course using dichotomized LLM RDoC scores reflecting a clinical need for treatment (see Table 3). Clinically relevant scores were highly prevalent: in LE narratives, 46 601 decedents (90.9%) evidenced at least 1 elevated domain and 62 139 (90.5%) evidenced at least 1 in CME narratives. Compared with decedents aged 65 years and older, younger decedents were more likely to show clinically relevant RDoC dysfunction after adjusting for confounding. This age-related pattern was consistent with 2 PHW-coded measures (mental health problem and alcohol or drug misuse) but not current depression, where depression prevalence was greatest in the oldest age group. Clinically relevant RDoC negative valence and arousal processes scores were more prevalent than PHW-coded measures. For example, among adults aged 65 years or older, 10 449 (83.4%) had clinically relevant RDoC scores in their CME narrative, but only 4775 (35.9%) had a PHW-coded mental health problem.

**Table 3.** Age Differences Across Mental Health Measures and Large Language Model (LLM)–Derived Research Domain Criteria Scores

Mental health indicator	Age, y						
	12-24		25-44		45-64		≥65^a^
	No. (%)	aOR (95% CI)^b^	No. (%)	aOR (95% CI)^b^	No. (%)	aOR (95% CI)^b^	No. (%)
NVDRS mental health measures							
No.	10 692	NA	25 226	NA	23 381	NA	13 286
Any mental health problem							
Yes	4614 (43.2)	1.49 (1.40-1.57)	11 687 (46.3)	1.64 (1.56-1.71)	11 114 (47.5)	1.58 (1.51-1.66)	4775 (35.9)
No or unknown	6078 (56.8)		13 539 (53.7)		12 267 (52.5)		8511 (64.1)
Alcohol or drug problem							
Yes	2142 (20.0)	2.31 (2.14-2.49)	9580 (38.0)	5.52 (5.17-5.89)	6887 (29.5)	3.68 (3.45-3.93)	1326 (10.0)
No or unknown	8550 (80.0)		15 646 (62.0)		16 494 (70.5)		11 960 (90.0)
Currently depressed							
Yes	2906 (27.2)	0.88 (0.83-0.94)	6524 (25.9)	0.8 (0.76-0.84)	6757 (28.9)	0.92 (0.87-0.96)	4069 (30.6)
No or unknown	7786 (72.8)		18 702 (74.1)		16 624 (71.1)		9217 (69.4)
Research Domain Criteria scores							
Law enforcement narratives							
Total No.	7541	NA	17 866	NA	16 430	NA	9403
Negative valence							
Moderate to extremely severe	6957 (92.3)	2.33 (2.08-2.61)	16 600 (92.9)	2.42 (2.21-2.64)	14 991 (91.2)	1.89 (1.74-2.06)	7984 (84.9)
None to mild	584 (7.7)		1266 (7.1)		1439 (8.8)		1419 (15.1)
Positive valence							
Moderate to extremely severe	2491 (33.0)	2.25 (2.08-2.43)	6796 (38.0)	2.74 (2.57-2.93)	5112 (31.1)	1.97 (1.85-2.11)	1676 (17.8)
None to mild	5050 (67.0)		11 070 (62.0)		11 318 (68.9)		7727 (82.2)
Social processes							
Moderate to extremely severe	2427 (32.2)	2.02 (1.87-2.19)	6333 (35.4)	2.26 (2.11-2.41)	5031 (30.6)	1.79 (1.68-1.91)	1784 (19.0)
None to mild	5114 (67.8)		11 533 (64.6)		11 399 (69.4)		7619 (81.0)
Arousal processes							
Moderate to extremely severe	4465 (59.2)	2.20 (2.05-2.37)	11 418 (63.9)	2.67 (2.52-2.83)	9430 (57.4)	1.98 (1.87-2.10)	3856 (41.0)
None to mild	3076 (40.8)		6448 (36.1)		7000 (42.6)		5547 (59.0)
Cognitive systems							
Moderate to extremely severe	1062 (14.1)	2.10 (1.88-2.35)	2719 (15.2)	2.28 (2.07-2.50)	2020 (12.3)	1.79 (1.62-1.97)	644 (6.8)
None to mild	6479 (85.9)		15 147 (84.8)		14 410 (87.7)		8759 (93.2)
Sensorimotor systems							
Moderate to extremely severe	199 (2.6)	1.52 (1.20-1.93)	461 (2.6)	1.61 (1.31-1.98)	317 (1.9)	1.29 (1.05-1.60)	133 (1.4)
None to mild	7342 (97.4)		17 405 (97.4)		16 113 (98.1)		9270 (98.6)
Any moderate to extremely severe domain score							
Yes	6970 (92.4)	2.36 (2.10-2.64)	16 624 (93.0)	2.44 (2.22-2.67)	15 012 (91.4)	1.9 (1.74-2.07)	7995 (85.0)
No	571 (7.6)		1242 (7.0)		1418 (8.6)		1408 (15.0)
Coroner or medical examiner narratives							
No.	10 142	NA	23 883	NA	22 095	NA	12 535
Negative valence							
Moderate to extremely severe	9274 (91.4)	2.18 (1.98-2.39)	22 138 (92.7)	2.58 (2.39-2.78)	20 166 (91.3)	2.03 (1.89-2.18)	10 423 (83.2)
None to mild	868 (8.6)		1745 (7.3)		1929 (8.7)		2112 (16.8)
Positive valence							
Moderate to extremely severe	3185 (31.4)	2.46 (2.30-2.64)	8966 (37.5)	3.24 (3.06-3.44)	6869 (31.1)	2.31 (2.18-2.45)	1957 (15.6)
None to mild	6957 (68.6)		14 917 (62.5)		15 226 (68.9)		10 578 (84.4)
Social processes							
Moderate to extremely severe	2970 (29.3)	2.11 (1.97-2.26)	7451 (31.2)	2.23 (2.11-2.37)	6130 (27.7)	1.81 (1.71-1.92)	2126 (17.0)
None to mild	7172 (70.7)		16 432 (68.8)		15 965 (72.3)		10 409 (83.0)
Arousal processes							
Moderate to extremely severe	5783 (57.0)	2.22 (2.09-2.36)	15 570 (65.2)	3.18 (3.03-3.35)	12 926 (58.5)	2.22 (2.11-2.33)	4885 (39.0)
None to mild	4359 (43.0)		8313 (34.8)		9169 (41.5)		7650 (61.0)
Cognitive systems							
Moderate to extremely severe	1455 (14.3)	2.59 (2.35-2.86)	3347 (14.0)	2.49 (2.29-2.72)	2606 (11.8)	1.98 (1.82-2.16)	776 (6.2)
None to mild	8687 (85.7)		20 536 (86.0)		19 489 (88.2)		11 759 (93.8)
Sensorimotor systems							
Moderate to extremely severe	243 (2.4)	1.82 (1.46-2.28)	568 (2.4)	1.93 (1.59-2.35)	386 (1.7)	1.54 (1.26-1.88)	141 (1.1)
None to mild	9899 (97.6)		23 315 (97.6)		21 709 (98.3)		12 394 (98.9)
Any moderate to extremely severe domain score							
Yes	9289 (91.6)	2.36 (2.10-2.64)	22 194 (92.9)	2.44 (2.22-2.67)	20 207 (91.5)	1.90 (1.74-2.07)	10 449 (83.4)
No	853 (8.4)		1689 (7.1)		1888 (8.5)		2086 (16.6)

Abbreviations: aOR, adjusted odds ratio; NA, not applicable; NVDRS, National Violent Death Reporting System.

^a^ Age 65 years and older served as the reference group.

^b^ aORs derived from multinomial logistic regression models modeling age as a function of mental health indicators after adjusting for sex, race and ethnicity, relationship status, military experience, geographic location, incident year, and narrative word length.

Finally, to evaluate whether including RDoC scores improved characterization of sex differences among suicide decedents, we used nested logistic regression equations to revisit the analysis in Table 4 (eTables 3, 4, and 5 in Supplement 1). Nearly all decedents evidenced high levels of negative valence dysfunctions, and the majority were experiencing disruption in arousal processes. Across most domains, female decedents showed clinically relevant dysfunctions more frequently than male decedents, except for sensorimotor processes. In all instances except 1, the addition of RDoC domain scores resulted in improvements in the fit of the regression models.

**Table 4.** Sex Differences in Large Language Model (LLM)–Derived Research Domain Criteria (RDoC) Scores

RDoC domain dysfunction scores	Sex			aOR (95% CI)^a^	LR χ^2^	*P* value
	Female (n = 10 129)	Male (n = 41 111)	Total (N = 51 240)			
Law enforcement narratives						
Negative valence						
Moderate to extremely severe	9412 (92.9)	37 120 (90.3)	46 532 (90.8)	1.16 (1.06-1.26)	10.77	.001
None to mild	717 (7.1)	3991 (9.7)	4708 (9.2)	1 [Reference]		
Positive valence						
Moderate to extremely severe	4080 (40.3)	11 995 (29.2)	16 075 (31.4)	1.45 (1.38-1.52)	203.75	<.001
None to mild	6049 (59.7)	29 116 (70.8)	35 165 (68.6)	1 [Reference]		
Social processes						
Moderate to extremely severe	3709 (36.6)	11 866 (28.9)	15 575 (30.4)	1.19 (1.13-1.25)	45.18	<.001
None to mild	6420 (63.4)	29 245 (71.1)	35 665 (69.6)	1 [Reference]		
Arousal processes						
Moderate to extremely severe	6546 (64.6)	22 623 (55.0)	29 169 (56.9)	1.25 (1.19-1.32)	73.78	<.001
None to mild	3583 (35.4)	18 488 (45.0)	22 071 (43.1)	1 [Reference]		
Cognitive systems						
Moderate to extremely severe	1526 (15.1)	4919 (12.0)	6445 (12.6)	0.98 (0.92-1.05)	0.22	.62
None to mild	8603 (84.9)	36 192 (88.0)	44 795 (87.4)	1 [Reference]		
Sensorimotor systems						
Moderate to extremely severe	149 (1.5)	961 (2.3)	1110 (2.2)	0.66 (0.56-0.79)	22.11	<.001
None to mild	9980 (98.5)	40 150 (97.7)	50 130 (97.8)	1 [Reference]		
Any moderate to extremely severe score						
Yes	9422 (93.0)	37 179 (90.4)	46 601 (90.9)	1.15 (1.05-1.26)	10.11	.002
No	707 (7.0)	3932 (9.6)	4639 (9.1)	1 [Reference]		
Coroner or medical examiner narratives						
Total No.	14 192	54 463	68 655	NA	NA	NA
Negative valence						
Moderate to extremely severe	13 298 (93.7)	48 703 (89.4)	62 001 (90.3)	1.30 (1.20-1.40)	45.56	<.001
None to mild	894 (6.3)	5760 (10.6)	6654 (9.7)	1 [Reference]		
Positive valence						
Moderate to extremely severe	5844 (41.2)	15 133 (27.8)	20 977 (30.6)	1.65 (1.58-1.73)	507.43	<.001
None to mild	8348 (58.8)	39 330 (72.2)	47 678 (69.4)	1 [Reference]		
Social processes						
Moderate to extremely severe	4833 (34.1)	13 844 (25.4)	18 677 (27.2)	1.25 (1.19-1.30)	96.41	<.001
None to mild	9359 (65.9)	40 619 (74.6)	49 978 (72.8)	1 [Reference]		
Arousal processes						
Moderate to extremely severe	9579 (67.5)	29 585 (54.3)	39 164 (57.0)	1.46 (1.40-1.53)	277.64	<.001
None to mild	4613 (32.5)	24 878 (45.7)	29 491 (43.0)	1 [Reference]		
Cognitive systems						
Moderate to extremely severe	2140 (15.1)	6044 (11.1)	8184 (11.9)	1.07 (1.01-1.13)	5.32	.02
None to mild	12 052 (84.9)	48 419 (88.9)	60 471 (88.1)	1 [Reference]		
Sensorimotor systems						
Moderate to extremely severe	192 (1.4)	1146 (2.1)	1338 (1.9)	0.71 (0.61-0.84)	18.82	<.001
None to mild	14 000 (98.6)	53 317 (97.9)	67 317 (98.1)	1 [Reference]		
Any moderate to extremely severe score						
Yes	13 320 (93.9)	48 819 (89.6)	62 139 (90.5)	1.30 (1.20-1.40)	44.35	<.001
No	872 (6.1)	5644 (10.4)	6516 (9.5)	1 [Reference]		

Abbreviations: aOR, adjusted odds ratio; LR χ^2^, likelihood ratio χ^2^ evaluating the increase in model fit with the addition of the RDoC dysfunction score; NA, not applicable; RDoC, research domain criteria.

^a^ aOR for each domain by type of narrative, adjusted logistic regression odds ratio derived from modeling female sex as a function of symptom scores after adjusting for demographic characteristics, mental health status measures, and narrative word length.

## Discussion

Suicide is closely linked to the human experience of intense and disruptive negative emotions: hopelessness, loss, anxiety, shame, anger, and agitation.^31^ For many, this is accompanied by a diagnosable mental disorder, but for others, it may represent a momentary crisis precipitated by life events.^19^ Using the RDoC framework with its emphasis on descriptive epidemiology rather than diagnostic prevalence, this study identified key dimensions of disrupted psychological functioning among specific subgroups of suicide decedents. Clinically relevant dysfunctions in the domains of negative valence, positive valence, social processes, and arousal processes were pervasive, with levels similar to those seen among psychiatric inpatients at admission.^15,20,21^

Our efforts to assess RDoC dysfunctions within this large database were facilitated by adopting methods developed for EHR processing (token-based RDoC scoring and zero-shot LLM RDoC scoring). Despite differences in purpose and structure between medical records and suicide narratives, both machine learning methods, even with a different LLM run locally, were effective in capturing dimensions of psychopathology present at the time of suicide. Further, robust sex and age differences observed were consistent with previous studies^9^ and clarify that the greatest burden of psychopathological dysregulation occurs among female decedents and those in early and middle adulthood. As medicine increasingly incorporates clinical decision support systems into health care settings, including supports targeting suicide prevention,^32^ findings from the current study underscore the need to consider broadly the panoply of dysregulation that can occur proximal to suicide. Such a system could be particularly useful for male patients who are less likely to receive formal mental health diagnoses, which are sometimes used to generate EHR alerts in clinical settings.^33^ Similarly, given the alarming rise in deaths by suicide in youths as early as 6 to 12 years,^1^ RDoC guidance from medical records to detect the potential for suicide could serve as a potential intervention in reducing deaths by suicide.^13^

### Limitations

Three study limitations warrant discussion. First, our use of an off-the-shelf LLM with zero-shot prompting had a proof-of-concept objective to demonstrate feasibility and ease in this approach. Improved accuracy of the RDoC scoring could have been obtained by using additional methods, such as providing scored examples along with the prompt (few-shot prompting), using techniques like retrieval augmented generation (RAG) to enhance model predictions, or comparing results across multiple LLMs, each trained differently. Without that effort, we nevertheless demonstrated that it is possible to apply a strategy developed for EHR text data to underutilized NVDRS text data. Second, NVDRS records are generated by multiple state-level coders using highly variable information sources. Narratives only summarize information available postmortem. For example, only about half of the incidents include toxicology reports, and many are missing LE reports.^9^ Although PHWs are thoroughly trained, there are likely a number of reporting biases generated by this approach. In our analyses, we attempted to control for some (eg, region of the country and year of death), but others remain, such as the propensity of various agencies, CME offices, or PHWs to be more or less thorough in their documentation. Also, evidence from 1 state office has indicated that the NVDRS likely undercounts mental health diagnoses and treatment episodes.^10^ To the extent that death narratives are similarly biased, we may have underestimated neurobehavioral disruptions. Third, while we tested the efficacy of this approach using only 2 demographic characteristics (sex and age), future work may benefit from investigating additional key exposures, such as race and ethnicity^8^ or sexual orientation,^34^ that may influence RDoC domain dysfunction. This could provide needed evidence of the generalizability of the current approach.

## Conclusions

Using the RDoC framework as a guide and emerging technology in machine learning methods, this study interrogated text data lying mostly fallow in the NVDRS database, and in doing so, uncovered at-risk levels of psychopathology among suicide decedents similar to those seen in psychiatric inpatients on admission. Prevalent dysfunction across the RDoC domains of negative valence, arousal processes, positive valence, and social processes underscores the intensity of psychological disruption that is common in suicide, especially among female and younger individuals. As health care increasingly mines EHR records for evidence that can be used to prevent suicide through developing early warning systems, combining both the RDoC approach demonstrated here and the tools of machine learning may facilitate this work.

## Notes

Supplement 1. **eFigure.** Diagram of Study Sample eTable 1. Description of Research Domain Criteria (RDoC) Domains According to the Large Language Model (LLM) eTable 2. Large Language Model Prompt Components eTable 3. Suicide Decedent RDoC Domain Scores Estimated by Token Density and the Large Language Model (LLM) for Law Enforcement (LE) and Coroner/Medical Examiner (CME) Narratives, 2020-2021 National Violent Death Reporting System (NVDRS) eTable 4. Sex Differences in Research Domain Criteria (RDoC) Token Density Scores, 2020-2021 National Violent Death Reporting System (NVDRS) eTable 5. Sex Differences in Large Language Model (LLM)-Derived Research Domain Criteria (RDoC) Scores in the 2020-2021 National Violent Death Reporting System (NVDRS)

Supplement 2. Data Sharing Statement
