# Supplementary material for: Research Domain Criteria and Deaths by Suicide in the National Violent Death Reporting System
Source: JAMA Netw Open. 2026 Mar 30;9(3):e264024. doi: 10.1001/jamanetworkopen.2026.4024 (PMC13036575; doi:10.1001/jamanetworkopen.2026.4024)
Supplement: Supplement 1. — eFigure. Diagram of Study Sample eTable 1. Description of Research Domain Criteria (RDoC) Domains According to the Large Language Model (LLM) eTable 2. Large Language Model Prompt Components eTable 3. Suicide Decedent RDoC Domain Scores Estimated by Token Density and the Large Language Model (LLM) for Law Enforcement (LE) and Coroner/Medical Examiner (CME) Narratives, 2020-2021 National Violent Death Reporting System (NVDRS) eTable 4. Sex Differences in Research Domain Criteria (RDoC) Token Density Scores, 2020-2021 National Violent Death Reporting System (NVDRS) eTable 5. Sex Differences in Large Language Model (LLM)-Derived Research Domain Criteria (RDoC) Scores in the 2020-2021 National Violent Death Reporting System (NVDRS) [file jamanetwopen-e264024-s001.pdf]

# Supplemental Online Content

Cochran SD, Chance C, Arseniev-Koehler A, Cuthbert B, Mays VM. Research domain criteria dysfunction among suicides in the national violent death reporting system. *JAMA Netw Open*. 2026;9(3):e264024. doi:10.1001/jamanetworkopen.2026.4024

eFigure. Diagram of Study Sample

eTable 1. Description of Research Domain Criteria (RDoC) Domains According to the Large Language Model (LLM)

eTable 2. Large Language Model Prompt Components

eTable 3. Suicide Decedent RDoC Domain Scores Estimated by Token Density and the Large Language Model (LLM) for Law Enforcement (LE) and Coroner/Medical Examiner (CME) Narratives, 2020-2021 National Violent Death Reporting System (NVDRS)

eTable 4. Sex Differences in Research Domain Criteria (RDoC) Token Density Scores, 2020-2021 National Violent Death Reporting System (NVDRS)

eTable 5. Sex Differences in Large Language Model (LLM)-Derived Research Domain Criteria (RDoC) Scores in the 2020-2021 National Violent Death Reporting System (NVDRS)

This supplemental material has been provided by the authors to give readers additional information about their work.

© 2026 Cochran SD et al. *JAMA Network Open*.

**eFigure.** Diagram of Study Sample

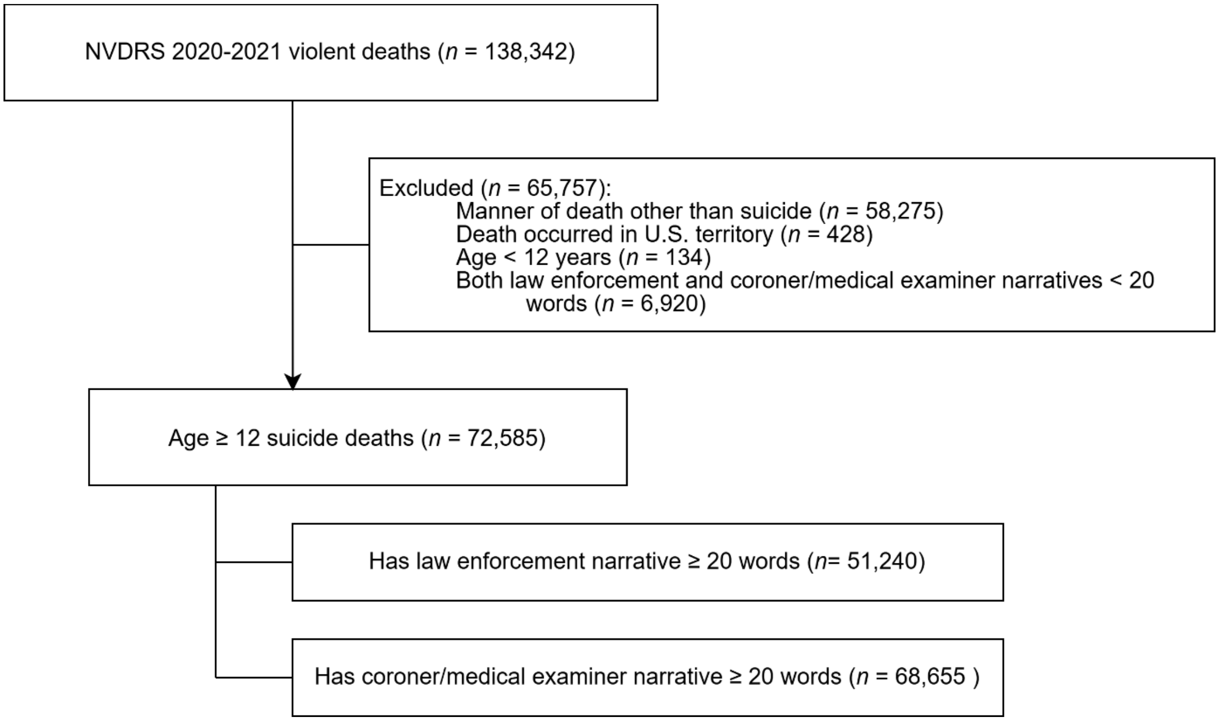


© 2026 Cochran SD et al. *JAMA Network Open*.

**eTable 1**. Description of Research Domain Criteria (RDoC) Domains According to the Large Language Model (LLM)

| Domain | Description |
|---|---|
| Negative valence systems | "The brain's response to aversive or unpleasant stimuli, such as pain, fear, or stress. It includes the neural circuits and processes that are involved in detecting and responding to threats or dangers." |
| Positive valence systems | "The brain's response to rewarding or pleasurable stimuli, such as food, sex, or social interaction. It includes the neural circuits and processes that are involved in seeking out and experiencing pleasure." |
| Social processes | "The brain's ability to interact with others, including social cognition, emotion regulation, and social behavior. It includes the neural circuits and processes that are involved in forming and maintaining social relationships." |
| Cognitive systems | "The brain's ability to process and interpret information, including attention, perception, memory, and decision-making. It includes the neural circuits and processes that are involved in thinking, problem-solving, and learning." |
| Arousal processes | "The brain's ability to regulate its own activity, including the regulation of attention, emotion, and motivation. It includes the neural circuits and processes that are involved in maintaining homeostasis and regulating the body's physiological responses." |
| Sensorimotor systems | "The brain's ability to interact with the environment through sensory and motor systems, including the processing of sensory information and the control of motor movements. It includes the neural circuits and processes that are involved in perceiving and acting on the world." |

**Note**. The LLM responded to this prompt: "Describe the Research Domain Criteria (RDoC) framework's 6 domains: Negative Valence Systems, Positive Valence Systems, Cognitive Systems, Social Processes, Arousal and Regulatory Systems, Sensorimotor Systems." Compare with the description given by NIH at https://www.nimh.nih.gov/research/research-funded-by-nimh/rdoc/definitions-of-the-rdoc-domains-and-constructs.

© 2026 Cochran SD et al. *JAMA Network Open*.

**eTable 2.** Large Language Model Prompt Components

| **Role** | **Prompt** |
|---|---|
| System | """You are a skilled NIMH Research Domain Criteria (RDoC) coder. Your task is to score a brief narrative from a post-mortem review of a suicide on a single RDoC domain. Use ONLY the information within this narrative and your skill. Remember that substance use can be reflected as a Positive Valence System symptom. Score this narrative on a 0–10 scale capturing the magnitude of documented domain symptoms: 0 if no symptoms are present or functioning is normal, 1–3 for mild symptoms, 4–6 for moderate symptoms requiring treatment, 7–9 for severe symptoms requiring hospitalization, and 10 if symptoms are extremely severe. Respond ONLY with this JSON format:<br>{<br> \"score\": <number or none><br>}<br>""" |
| User | domain = [<br> "Negative Valence Systems", "Positive Valence Systems", "Cognitive Systems",<br> "Social Processes", "Arousal and Regulatory Systems", "Sensorimotor Systems"<br> ]<br><br>"You are now scoring the **{domain}** domain ONLY. Narrative: **{narrative}** " |

© 2026 Cochran SD et al. *JAMA Network Open*.

**eTable 3. Suicide Decedent RDoC Domain Scores Estimated by Token Density and the Large Language Model (LLM) for Law Enforcement (LE) and Coroner/Medical Examiner (CME) Narratives, 2020-2021 National Violent Death Reporting System (NVDRS)**

| RDoC Domain | RDoC Tokens[1] Mean (SD) | LLM RDoC Scores[2] Mean (SD) | Perplexity Scores[3] Mean (SD) | Kendall's Tau-b |
|---|---|---|---|---|
| **LE narrative (n = 51,240)** | | | | |
| Negative valence | 2.35 (2.23) | 7.32 (2.67) | 2.28 (0.75) | 0.06 |
| Positive valence | 2.73 (3.81) | 2.11 (2.95) | 2.11 (1.10) | 0.13 |
| Social processes | 0.42 (1.18) | 2.06 (2.84) | 2.03 (1.02) | 0.07 |
| Arousal processes | 1.23 (1.59) | 4.01 (3.50) | 2.51 (1.08) | 0.07 |
| Cognitive systems | 0.86 (1.33) | 0.80 (2.04) | 1.52 (0.77) | 0.06 |
| Sensorimotor systems[1] | --- | 0.21 (1.41) | 1.06 (0.25) | |
| **CME narrative (n = 68,655 )** | | | | |
| Negative valence | 2.44 (2.19) | 7.17 (2.66) | 2.33 (0.76) | 0.10 |
| Positive valence | 2.85 (3.78) | 2.15 (2.93) | 2.24 (1.15) | 0.30 |
| Social processes | 0.44 (1.21) | 1.91 (2.77) | 2.06 (1.04) | 0.18 |
| Arousal processes | 1.27 (1.60) | 4.01 (3.42) | 2.60 (1.09) | 0.15 |
| Cognitive systems | 0.92 (1.37) | 0.76 (1.99) | 1.53 (0.78) | 0.14 |
| Sensorimotor systems[1] | --- | 0.19 (1.34) | 1.05 (0.23) | |

**Note.** RDoC = Research Domain Criteria; LLM = large language model; NVDRS = National Violent Death Reporting System; Mean law enforcement (LE) narrative word length = 156.4 (SD = 110.8); Mean coroner/medical (CME) examiner narrative word length = 152.1 (SD = 100.8); Kendall's Tau-b compares RDoC token scores with LLM RDoC scores within each domain, all P's < 0.001.

[1]RDoC tokens per 100 narrative tokens (McCoy, 2018, DOI: 10.1016/j.xjmad.2024.100079). Sensorimotor domain is not estimated.

[2]Large language model (LLM) RDoC scores ranged from 0-10; 0 = no symptoms; 1-3 mild symptoms; 4-6 moderate symptoms requiring treatment; 7-9 severe symptoms requiring hospitalization; 10 = severe symptoms.

[3]Perplexity score estimated from the exponent of the negative log probability of the assigned LLM score.

© 2026 Cochran SD et al. *JAMA Network Open*.

**eTable 4. Sex Differences in Research Domain Criteria (RDoC) Token Density Scores, 2020-2021 National Violent Death Reporting System (NVDRS)**

| RDoC Domain dysfunctions | Sex | | AOR (95% CI) | LR $\chi^2$ | P |
|---|---|---|---|---|---|
| | Female | Male | | | |
| **LE narratives** | | | | | |
| No: | 10,129 | 41,111 | | | |
| Negative valence, mean (SD) | 2.4 (2.4) | 2.4 (2.2) | 0.98 [0.97-0.99] | 21.78 | <0.001 |
| Positive valence, mean (SD) | 3.1 (4.1) | 2.6 (3.7) | 1.02 [1.02-1.03] | 59.62 | <0.001 |
| Social Processes, mean (SD) | 0.4 (1.2) | 0.4 (1.2) | 0.99 [0.97-1.01] | 1.82 | 0.18 |
| Arousal processes, mean (SD) | 1.4 (1.7) | 1.2 (1.6) | 1.02 [1.01-1.04] | 11.47 | 0.001 |
| Cognitive systems, mean (SD) | 0.9 (1.4) | 0.8 (1.3) | 1.00 [0.98-1.01] | 0.29 | 0.59 |
| **CME narratives** | | | | | |
| No: | 14,192 | 54,463 | | | |
| Negative valence, mean (SD) | 2.4 (2.3) | 2.4 (2.2) | 0.97 [0.96-0.98] | 49.84 | <0.001 |
| Positive valence, mean (SD) | 3.2 (4.0) | 2.8 (3.7) | 1.03 [1.02-1.03] | 82.80 | <0.001 |
| Social Processes, mean (SD) | 0.4 (1.2) | 0.4 (1.2) | 0.98 [0.96-0.99] | 6.91 | 0.001 |
| Arousal processes, mean (SD) | 1.4 (1.7) | 1.2 (1.6) | 1.02 [1.01-1.04] | 14.27 | <0.001 |
| Cognitive systems, mean (SD) | 1.0 (1.4) | 0.9 (1.4) | 1.00 [0.98-1.01] | 0.43 | 0.51 |

**Note**. RDoC = Research Domain Criteria; Token density = domain dysfunction tokens/100 narrative tokens; AOR = Adjusted odds ratio associated with female sex from the RDoC token density scores after adjusting for demographic characteristics and mental health status indicators. LR $\chi^2$ = Likelihood Ratio Chi-square test evaluates the increase in model fit with the addition of the RDoC token score; P = probability that the LR $\chi^2$ value is consistent with chance variation.

© 2026 Cochran SD et al. *JAMA Network Open*.

**eTable 5. Sex Differences in Large Language Model (LLM)-Derived Research Domain Criteria (RDoC) Scores in the 2020-2021 National Violent Death Reporting System (NVDRS)**

| | Sex | | | | |
|---|---|---|---|---|---|
| RDoC Domain dysfunction | Female | Male | AOR (95% CI) | LR $\chi^2$ | P |
| **LE narratives** | | | | | |
| No: | 10,129 | 41,111 | | | |
| Negative valence, mean (SD) | 7.4 (2.4) | 7.3 (2.7) | 1.01 [1.00-1.02] | 2.05 | 0.15 |
| Positive valence, mean (SD) | 2.6 (3.1) | 2.0 (2.9) | 1.06 [1.05-1.07] | 198.15 | <0.001 |
| Social Processes, mean (SD) | 2.4 (3.0) | 2.0 (2.8) | 1.03 [1.02-1.04] | 42.69 | <0.001 |
| Arousal processes, mean (SD) | 4.4 (3.4) | 3.9 (3.5) | 1.02 [1.02-1.03] | 42.04 | <0.001 |
| Cognitive systems, mean (SD) | 0.9 (2.2) | 0.8 (2.0) | 0.99 [0.98-1.01] | 0.93 | 0.34 |
| Sensorimotor systems, mean (SD) | 0.1 (1.2) | 0.2 (1.5) | 0.96 [0.94-0.97] | 0.43 | 0.51 |
| **CME narratives** | | | | | |
| No: | 14,192 | 54,463 | | | |
| Negative valence, mean (SD) | 7.5 (2.3) | 7.1 (2.8) | 1.04 [1.03-1.05] | 79.82 | <0.001 |
| Positive valence, mean (SD) | 2.8 (3.2) | 2.0 (2.8) | 1.09 [1.08-1.10] | 577.27 | <0.001 |
| Social Processes, mean (SD) | 2.4 (3.0) | 1.8 (2.7) | 1.03 [1.03-1.04] | 83.47 | <0.001 |
| Arousal processes, mean (SD) | 4.7 (3.3) | 3.8 (3.4) | 1.05 [1.05-1.06] | 249.66 | <0.001 |
| Cognitive systems, mean (SD) | 1.0 (2.2) | 0.7 (1.9) | 1.01 [1.00-1.02] | 5.27 | 0.02 |
| Sensorimotor systems, mean (SD) | 0.1 (1.1) | 0.2 (1.3) | 0.97 [0.95-0.98] | 18.28 | <0.001 |

**Note**. LLM = large language model; NVDRS = National Violent Death Reporting System; RDoC = Research Domain Criteria; AOR = Adjusted odds ratio associated with female sex from the LLM RDoC scores after adjusting for demographic characteristics, mental health status indicators, and narrative word count; LR $\chi^2$ = Likelihood Ratio Chi-square test evaluates the increase in model fit with the addition of the LLM RDoC score; P = probability that the LR $\chi^2$ value is consistent with chance variation.

© 2026 Cochran SD et al. *JAMA Network Open*.
