# Supplementary material for: Research Domain Criteria and Deaths by Suicide in the National Violent Death Reporting System
Source: JAMA Netw Open. 2026 Mar 30;9(3):e264024. doi: 10.1001/jamanetworkopen.2026.4024 (PMC13036575; doi:10.1001/jamanetworkopen.2026.4024)
Supplement: Supplement 2. — Data Sharing Statement [file jamanetwopen-e264024-s002.pdf]

Data Sharing Statement

Cochran. Research Domain Criteria and Deaths by Suicide in the National Violent Death Reporting System. *JAMA Netw Open*. Published March 30, 2026. doi:10.1001/jamanetworkopen.2026.4024

Data
Data available: No

Additional Information
Explanation for why data not available: This is a restricted dataset obtainable from the CDC subject to their approval. We did not collect any additional data.
